# Crystallization and Orientation Modulation Enable Highly Efficient Doctor-Bladed Perovskite Solar Cells

**DOI:** 10.1007/s40820-023-01138-x

**Published:** 2023-06-29

**Authors:** Jianhui Chang, Erming Feng, Hengyue Li, Yang Ding, Caoyu Long, Yuanji Gao, Yingguo Yang, Chenyi Yi, Zijian Zheng, Junliang Yang

**Affiliations:** 1 Hunan Key Laboratory of Nanophotonics and Devices, School of Physics and Electronics, Central South University, Changsha, 410083 Hunan People’s Republic of China; 2 Shanghai Synchrotron Radiation Facility (SSRF), Shanghai Advanced Research Institute, Chinese Academy of Sciences, Shanghai, 201204 People’s Republic of China; 3 Department of Electrical Engineering, Tsinghua University, Beijing, 100084 People’s Republic of China; 4 Department of Applied Biology and Chemical Technology, Faculty of Science, The Hong Kong Polytechnic University, Hong Kong, 999077 People’s Republic of China

**Keywords:** Crystallization regulation, Orientation modulation, Perovskite solar cells, Doctor-blading, Ambient condition

## Abstract

With the rapid rise in perovskite solar cells (PSCs) performance, it is imperative to develop scalable fabrication techniques to accelerate potential commercialization. However, the power conversion efficiencies (PCEs) of PSCs fabricated via scalable two-step sequential deposition lag far behind the state-of-the-art spin-coated ones. Herein, the additive methylammonium chloride (MACl) is introduced to modulate the crystallization and orientation of a two-step sequential doctor-bladed perovskite film in ambient conditions. MACl can significantly improve perovskite film quality and increase grain size and crystallinity, thus decreasing trap density and suppressing nonradiative recombination. Meanwhile, MACl also promotes the preferred face-up orientation of the (100) plane of perovskite film, which is more conducive to the transport and collection of carriers, thereby significantly improving the fill factor. As a result, a champion PCE of 23.14% and excellent long-term stability are achieved for PSCs based on the structure of ITO/SnO_2_/FA_1-*x*_MA_*x*_Pb(I_1-*y*_Br_*y*_)_3_/Spiro-OMeTAD/Ag. The superior PCEs of 21.20% and 17.54% are achieved for 1.03 cm^2^ PSC and 10.93 cm^2^ mini-module, respectively. These results represent substantial progress in large-scale two-step sequential deposition of high-performance PSCs for practical applications.

**Supplementary Information:**

The online version contains supplementary material available at 10.1007/s40820-023-01138-x.

## Highlights

- High quality and strongly oriented perovskite films are prepared by two step doctor blading in air.
- Methylammonium chloride induced low dimensional intermediate phase regulates the crystallization and orientation of the perovskite.
- Two step doctor bladed perovskite solar cells achieve a power conversion efficiency (PCE) of 23.14%, while 1.03 cm^2^ PSCs and 10.93 cm^2^ mini modules achieve PCEs of 21.20% and 17.54%, respectively.

**Supplementary Information:**

The online version contains supplementary material available at 10.1007/s40820-023-01138-x.

## Graphical abstract

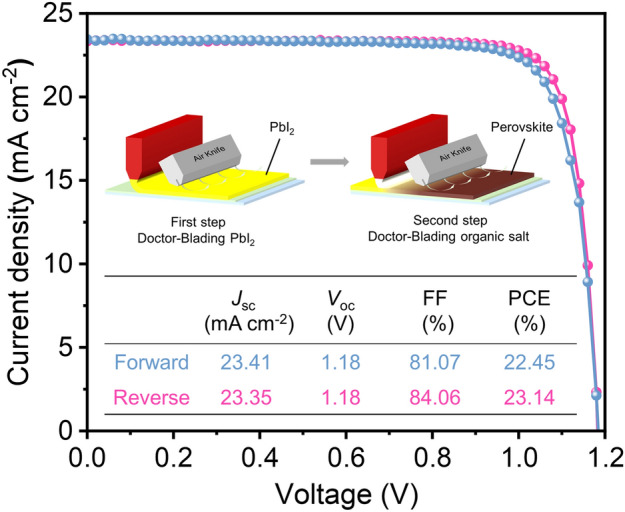

## Introduction

Hybrid organic–inorganic metal halide perovskite materials possess excellent optoelectronic properties, including high absorption coefficient, long carrier diffusion length, and low trap density, leading to the certified power conversion efficiency (PCE) of perovskite solar cells (PSCs) to 25.7% [1–4]. However, most reported high-efficiency PSCs are fabricated via laboratory-scale spin-coating deposition. It is essential to develop scalable and large-area fabrication techniques for processing efficient and stale PSCs for matching with industrial commercialization applications.

Doctor-blading has been demonstrated as a convenient, effective, and low-cost technique for upscaling perovskite films and PSC devices [5–8]. It is significant to bring this emerging technology from the laboratory to the marketplace. The one-step doctor-blading technology combined with the strategies of composition engineering, solvent engineering, surfactant engineering, etc., could achieve uniform and large-area perovskite films, thereby improving the PCE and stability of PSCs [9–12]. However, it is very challenging to control the nucleation and growth processes during the one-step deposition of perovskite films in an air environment, which significantly increases the complexity of controlling the morphology of large-area films [13, 14].

Two-step sequential deposition using organic salts to react with lead halide, skipping the most difficult process of uncontrollable nucleation of perovskite film in one-step process, resulting in the PCE of the two-step spin-coated PSCs exceeding 25.6% [15]. However, the performance of two-step doctor-bladed PSCs still falls far short of the state-of-the-art two-step spin-coated ones. In the two-step process, the high-quality PbI_2_ layer is the key to producing excellent perovskite film [16–21]. Matteocci et al. added methylammonium iodide (MAI) into the PbI_2_ solution to optimize the morphology of the PbI_2_ film and the conversion step from PbI_2_ to perovskite, suppressing the crystal defects [22]. Particularly, the dense PbI_2_ film would hinder the permeation and diffusion of organic salts and inhibit the conversion of PbI_2_ to perovskite, while porous PbI_2_ can well react with organic salts [23–25]. Zhang et al. produced a continuous and regular nano-structure PbI_2_ film through doctor-blading by incorporating high boiling point and low coordination 4-*tert*-butylpyridine (TBP) into the PbI_2_ solution as a pore-guiding additive, and a champion PCE of 20.49% was achieved by further adding the appropriate amount of perovskite crystal seeds into PbI_2_ [26]. Wen et al. controlled the crystallization process of PbI_2_ by introducing THTO (tetrahydrothiophene 1-oxide) to form a PbI_2_–THTO complex to obtain an ideal nanochannel structure and finally received a PCE of 22.77%, which is the PCE record so far for two-step sequentially doctor-bladed PSCs [27].

This work demonstrates high-quality perovskite film fabricated via two-step sequential doctor-blading. The presence of MACl can induce the direct transformation of precursors into *α*-phase perovskite, enhancing the crystalline quality and grain size, reducing trap density, and suppressing the nonradiative recombination. Moreover, utilizing MACl as an additional agent can achieve the orientation growth of perovskite crystals, facilitating efficient charge transport and boosting the fill factor. Consequently, 0.064 cm^2^ PSCs exhibit the champion PCEs of 23.14%, representing the PCE records for two-step doctor-bladed PSCs. Meanwhile, 1.03 cm^2^ PSCs and 10.93 cm^2^ mini-modules fabricated through this strategy achieve a PCE of 21.20% and 17.54%, respectively. Furthermore, the unencapsulated devices maintain 80% of their initial PCEs after 70 days of storage in ambient conditions with a humidity of 30% RH. These results demonstrate the exceptional potential of two-step sequential doctor-blading deposition in manufacturing high-performance large-area PSCs.

## Experimental Sections

### Material

SnO_2_ precursor (Alfa Aesar, 15% in H_2_O colloidal dispersion) was purchased from Alfa Aesar. PbI_2_ was purchased from Advance Election Technology Co., Ltd. HC(NH_2_)_2_I (FAI, 99.5%), CH_3_NH_3_Cl (MACl, 99.5%), CH_3_NH_3_Br (MABr, 99.5%), octyl ammonium iodide (OAI, 99.5%), and 2,2′,7,7′-tetrakis[*N,N*-di(4-methoxyphenyl)amino]-9,9′-spirobifluorene (Spiro-OMeTAD, 99.5%) were purchased from Xi'an Polymer Light Technology Corp. Isopropanol (IPA, 99.5%), *N,N*-dimethylformamide (DMF, 99.8%), chlorobenzene (CB, 99.8%), 4-tert-butylpyridine (TBP, 99.9%), and acetonitrile (ACN, 99.95%) were purchased from Sigma-Aldrich. All materials were used directly without any purification.

### Device Fabrication

The glass/ITO was cleaned ultrasonically with detergent, deionized water, and isopropanol for 20 min, sequentially, then dried, and treated with UV–ozone for 25 min. The 2.67% SnO_2_ colloidal solution diluted by deionized water was spin-coated on glass/ITO substrate at a speed of 3,000 rpm for 30 s, followed by annealing at 150 °C for 30 min. Before the deposition of perovskite films, the samples were treated with UV–ozone for 20 min. After UV treatment, the perovskite films were deposited onto the achieved SnO_2_ substrates via the two-step doctor-blading sequential process. 1.0 mM PbI_2_ was dissolved in 1 mL mixed solvent (DMF/TBP = 9:1) and stirred at 75 °C for overnight. First, a certain amount of PbI_2_ precursor solution was dripped into the gap (about 300 μm) between the blade knife and substrate; the horizontal movement speed of the blade knife is 15 mm s^−1^. The wet films were dried immediately with N_2_ air knife and then annealed at 70 °C for 30 min to obliterate the solvent. After the substrate was cooled down to room temperature, the organic salt solutions of FAI: MABr (80 mg:8 mg) with different ratios MACl (0, 10, 20, and 30 wt% to FAI) were bladed onto the PbI_2_ films, dried using N_2_ air knife, and then annealed at 150 °C for 20 min for perovskite crystallization. After cooling, the perovskite films were transferred into the N_2_ glove box. For perovskite films requiring OAI modifications, a 5-mg mL^−1^ OAI/IPA solution was spun onto the doctor-bladed perovskite films, followed by annealing at 100 °C for 10 min. For the hole transport layer, the Spiro-OMeTAD solution, 72.3 mg of Spiro-OMeTAD with Li-TFSI (17.5 μL from a 520-mg mL^−1^ stock solution in ACN) and TBP (28.8 μL) as dopants into 1 ml of CB, was spin-coated onto the perovskite layer at 4,000 rpm for 30 s. Finally, 100-nm-thick Ag counterelectrode was deposited via a thermal evaporation system at a pressure of less than 5 × 10^−4^ Pa. All the doctor-blading process was implemented in ambient air with humidity around 40%–50% RH.

### Characterization

The X-ray diffraction (XRD) patterns of the perovskite thin films were obtained on a SmartLab SE system (Rigaku, Japan) using Cu K*α* radiation (*λ* = 1.5405 Å) as the X-ray source. The GIWAXS was performed at BL17B1 beamline of SSRF using the X-ray energy of 10 keV. The scanning electron microscopy images were acquired using a field-emission scanning electron microscope (FEI Helios Nanolab 600i SEM, USA). The ultraviolet–visible (UV–Vis) spectrophotometer (Puxi, T9, China) was used to characterize the absorption properties of perovskite films. Steady-state photoluminescence (PL) and time-resolved PL spectra were measured by a FLS980 (Edinburgh Inc.) spectrometer. Current density–voltage (*J–V*) characteristics of the devices were measured from –0.1 to 1.2 V with a 50-ms scanning delay by a digital source meter (Keithley, model 2400). The devices were illuminated by a xenon-lamp-based solar simulator (Enlitech SS-X, AM 1.5G), calibrated by a standard silicon solar cell with a light intensity of 1 Sun.

## Results and Discussion

### Characterization of Perovskite Films

**Fig. 1:**
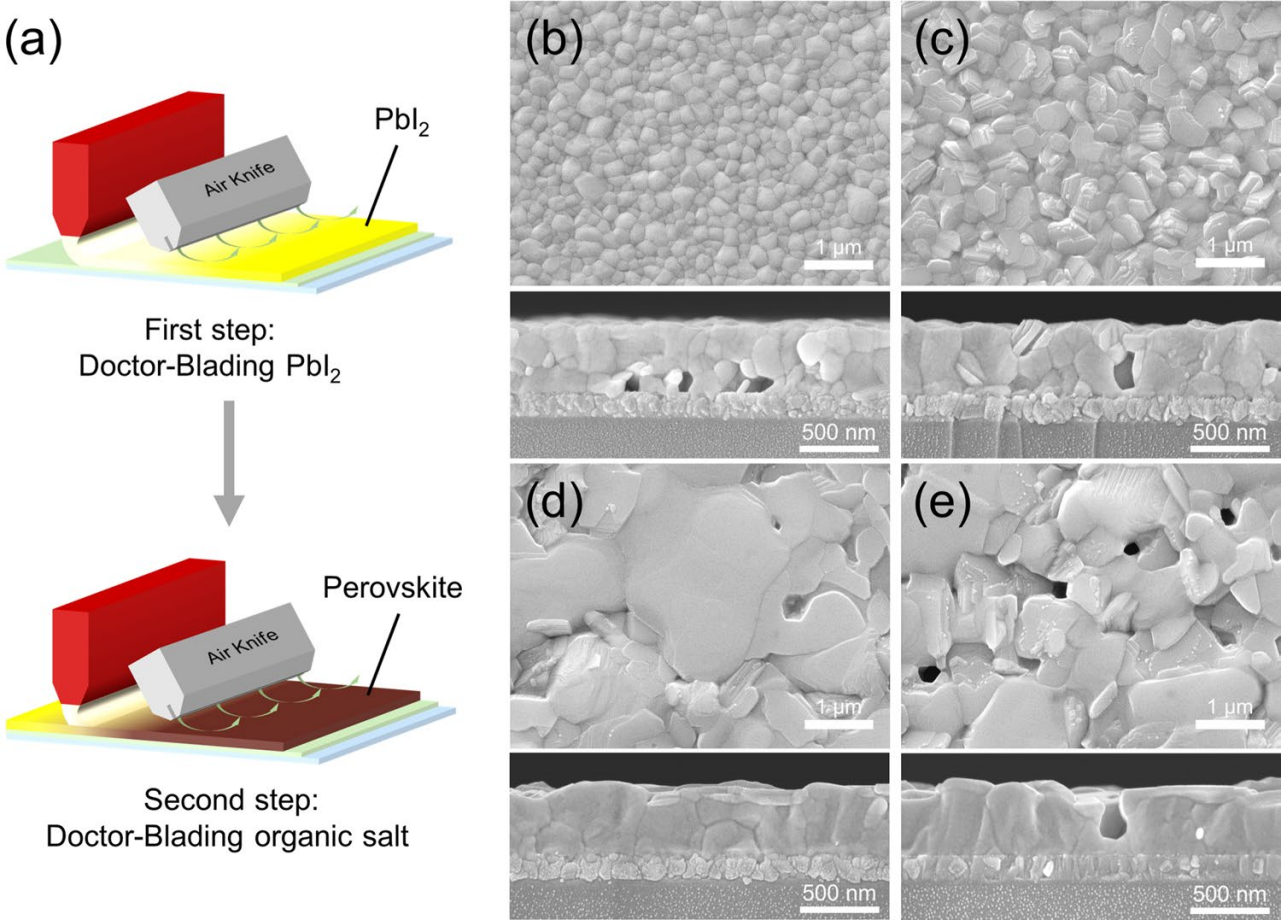
**a** Schematic illustration of the two-step sequential doctor-bladed deposition. Top-view (top) and cross-sectional (bottom) SEM images for perovskite films fabricated via two-step doctor-blading with the different ratios of MACl for **b** MACl-0, **c** MACl-10%, **d** MACl-20%, and **e** MACl-30%

Figure 1a demonstrates the procedure of the two-step sequential doctor-blading deposition of perovskite films. First, the PbI_2_ solution is doctor-bladed on ITO/SnO_2_ substrate and dried with N_2_ air knife, followed by annealing and crystallization at 70 °C. In order to ensure enough channels in the PbI_2_ film for the easy penetration of organic amine salt in the second step, a small amount of 4-tert-butylpyridine (TBP) is added to the PbI_2_ solution to control the morphology. The non-volatile TBP does not easily volatilize in the doctor-blading process due to a high boiling point and can escape during the subsequent annealing procedure because of its low solubility with the PbI_2_, causing holes in the PbI_2_ film [26]. As anticipated, the top-view scanning electron microscopy (SEM) image shows that the PbI_2_ film demonstrates a uniform distribution of pores, facilitating the organic salts to diffuse into the PbI_2_ film and induce crystal growth, as shown in Fig. S1. Subsequently, the organic salt solution is doctor-bladed atop the PbI_2_ film and dried using N_2_ air knife. The pre-dried film is annealed at 150 °C to aid the growth of the perovskite grains. It is shown that the different MACl contents (the mass ratio of MACl to FAI denoted as 0, 10%, 20%, and 30%) strongly influence the morphology of the final perovskite film. Figure 1b–e shows the top-view and cross-sectional SEM images of perovskites fabricated with the different contents of MACl after annealing. A more extensive range of surface morphologies and particle size statistics are shown in Fig. S2. The MACl-0 perovskite film consists of a stack of tiny grains with cavities at the base. The MACl-10% perovskite film exhibits grains with a larger size, but holes are still hidden deep within the film. The grain size of perovskite film continues to increase as the proportion of MACl increases. The grains of MACl-20% perovskite film have an average size of more than 2 μm and are closely aligned in the vertical direction with no cavities, and grain boundaries are significantly reduced, facilitating carrier transport. Further increasing the proportion of MACl to 30%, uniformly distributed holes appear on the perovskite film, and the depth of these cavities is almost equal to the thickness of the film. The escape of excess MACl causes the appearance of holes during the heating process. The holes cause an increase in the contact probability between the upper and back electrodes in final PSCs.

**Fig. 2:**
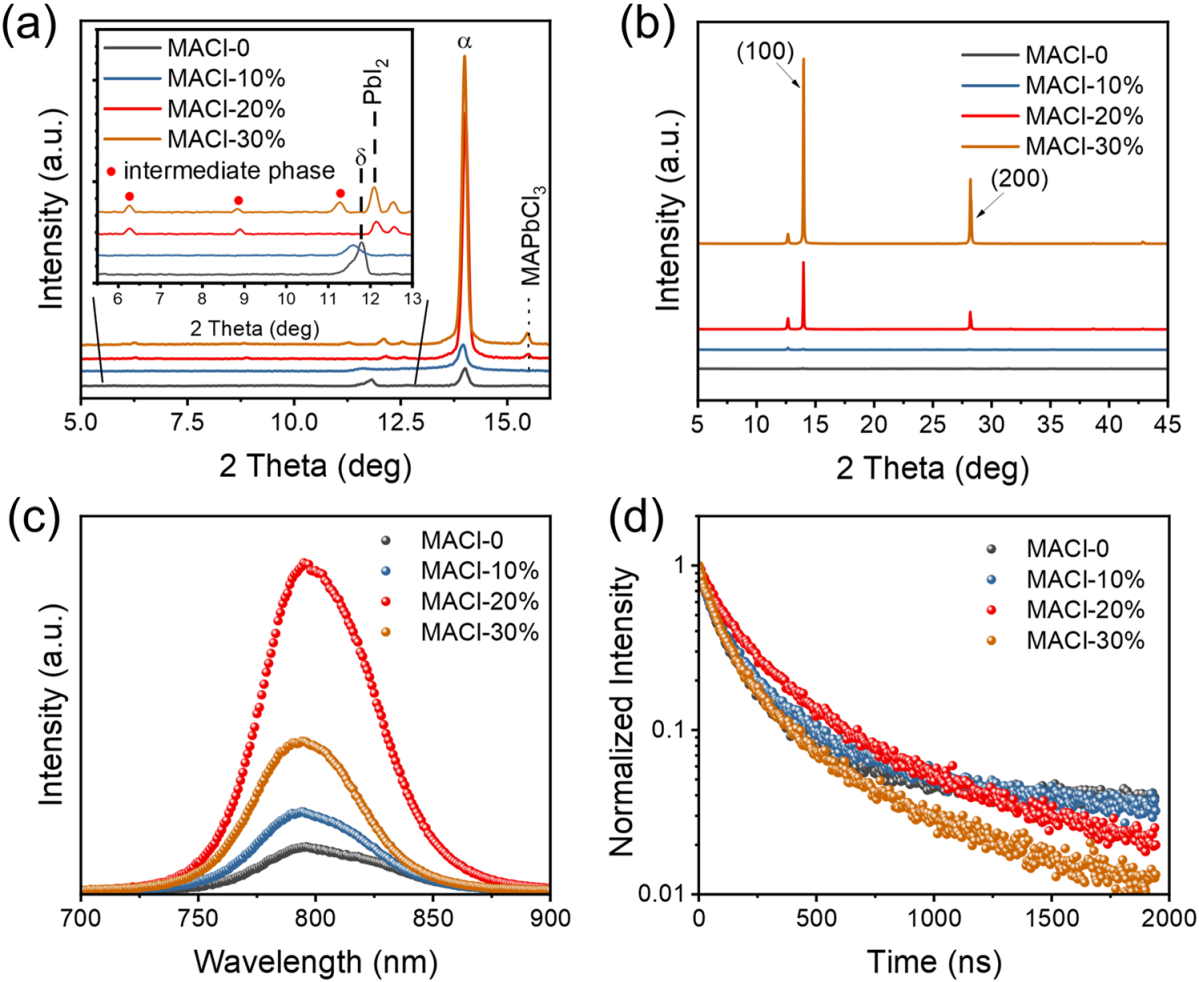
Characterization of perovskite films fabricated via two-step doctor-blading. **a** XRD patterns of wet pristine perovskite films. **b** XRD patterns of perovskite films after annealing. **c** Steady-state PL emission spectra. **d** Time-resolved PL decay curves

In addition to the surface morphology variation, the crystallinity varies with the MACl ratio. Figure 2a shows the XRD patterns of the unannealed precursor films. In MACl-0 and MACl-10%, an obvious *δ*-phase appeared, and the intensity of the *α*-phase was weak. After adding a sufficient amount of MACl, MACl-20% and MACl-30% exhibit a vital *α*-phase diffraction peak accompanied by faint PbI_2_, which indicates that MACl can significantly reduce the formation energy of the *α*-phase and is related to the amount of incorporation [28, 29]. The unannealed MACl-20% precursor film shows two low-angle diffraction peaks at 6.3° and 8.9°, and the MACl-30% precursor film shows three low-angle diffraction peaks at 6.3°, 8.9°, and 11.3°. Low-angle diffraction peaks imply that a sufficient MACl can induce low-dimensional intermediate phases. It cannot find low-dimensional intermediate phases in the precursor film where FACl or MAI replaced MACl, as shown in Fig. S3a. FACl or MAI-added perovskite films have low crystallinity and weak orientation after annealing, as shown in Fig. S3b. In contrast, an organic salt solution of only MACl is deposited on the PbI_2_ film, faint *α*-phase perovskite appears, and no low-dimensional phase appears, as shown in Fig. S4a. After annealing, the *α*-phase disappears. There is only PbI_2_, and the intensity increases, obviously. The full width at half maximum (FWHM) of the (001) peak associated with PbI_2_ decreases by two times, which indicates that the degree of crystallinity increases, as shown in Fig. S4b. Concurrently, it results in the decline of the (011)/(001) peak intensity ratio (from 0.175 to 0.051), indicating a stronger orientation on the *c*-axis, which may be related to the formation of the MACl intercalation PbI_2_ complex and decomposition.

During the annealing process, the low-dimensional intermediate phase disappears, the crystallinity of the perovskite phase increases, and it exhibits a significant (100) orientation, as shown in Fig. S5. Low-dimensional intermediate phases can compete with FA^+^, which can retard the crystallization process of perovskite and support the formation of micron-sized crystalline grains. More meaningfulness is that the low-dimensional intermediate phase possibly provides a template for the later nucleation and growth of perovskite, eventually leads to the highly oriented crystalline growth of perovskite in the vertical direction [30, 31].

Figures 2b and S6a show that each annealed perovskite film is a pure *α*-phase perovskite. The increase in diffraction intensity and the decrease in the FWHM of the peak at ~ 13.9° (Fig. S6b) demonstrate that the crystallinity and grain size are strongly enhanced with the increase in MACl ratio, which agrees well with the large size grain observed in SEM images. Nevertheless, too much MACl will destroy the integrity of the perovskite film, as shown in Fig. 1. Furthermore, it is found that the exact position of the XRD peaks associated with the (100) and (200) crystal planes for different samples shifts to a larger angle as the MACl ratio increases (Fig. S6c). A larger diffraction peak angle means that the perovskite lattice parameters become smaller because more small-sized MA cations enter the lattice during the growth of perovskite crystals. It should be noted that the PbI_2_ diffraction peaks are not obviously found in the final MACl-0 film (Fig. S7), which indicates that PbI_2_ is not in excess and FAI/MABr can react entirely with PbI_2_. In the case of MACl-10%, the diffraction intensity of PbI_2_ is much stronger than that of perovskite. For MACl-20% and MACl-30%, the intensity of the (100) planes of perovskite is much stronger than that of PbI_2_. It suggests that the residue of PbI_2_ is related to the decomposition of the intermediate phase. More MACl competes with FA^+^ to generate more intermediate phases in the initial reaction stage of organic amine salt and PbI_2_. During subsequent annealing, the intermediate phase decomposes and releases PbI_2_. The drastic changes in morphology and crystallinity suggest that MACl strongly modulates the nucleation and growth of the perovskite films prepared by the two-step doctor-blading.

The UV–Vis spectra (Fig. S8a) show that light absorption is enhanced due to the obvious the increased grain size and crystallinity. But each perovskite film has a similar bandgap (*E*_g_) of ~ 1.56 eV with subtle differences estimated by Tauc plots (Fig. S8b and Table S1). The steady-state PL spectra also exhibit the same pattern, with the position of the emission peak remaining essentially unchanged after adding MACl (Fig. 2c). Meanwhile, compared with reference perovskite film, high-quality MACl perovskite films bring a higher PL intensity, which means a lower trap-state density and less nonradiative recombination loss. Defects within perovskite films act as carrier trap centers where the energy is lost through nonradiative recombination pathways. The time-resolved photoluminescence (TRPL) decay profiles are performed to evaluate the charge-carrier lifetimes, as shown in Fig. 2d. The decay curves are fitted by Eq. (1) [32]:

1 $$y = y_{0} + A_{1} e^{{ - x/\tau_{1} }} + A_{2} e^{{ - x/\tau_{2} }} + A_{3} e^{{ - x/\tau_{3} }}$$

where *A*_1,_ *A*_2,_ and *A*_3_ are the time-independent coefficients of amplitude fraction and *τ*_1_, *τ*_2,_ and *τ*_3_ are the fast decay time, intermediate decay time, and slow decay time, respectively. The fitted decay times of corresponding perovskite films are summarized in Table S2. The MACl-20% film exhibits a longer average carrier lifetime (342.78 ns) than the MACl-0 film (205.32 ns). The MACl-30% film provides a lower average carrier lifetime (281.22 ns) than MACl-20%, caused by the holes in the film introducing more defects. The longer carrier lifetime indicates that the nonradiative composite loss is significantly suppressed due to a reduction in defect-mediated bulk or surface recombination, which is inextricably linked to the increased crystalline strength of the perovskite film, the increased grain size, and the reduced grain boundaries.

### Orientation and Crystallization Mechanism

**Fig. 3:**
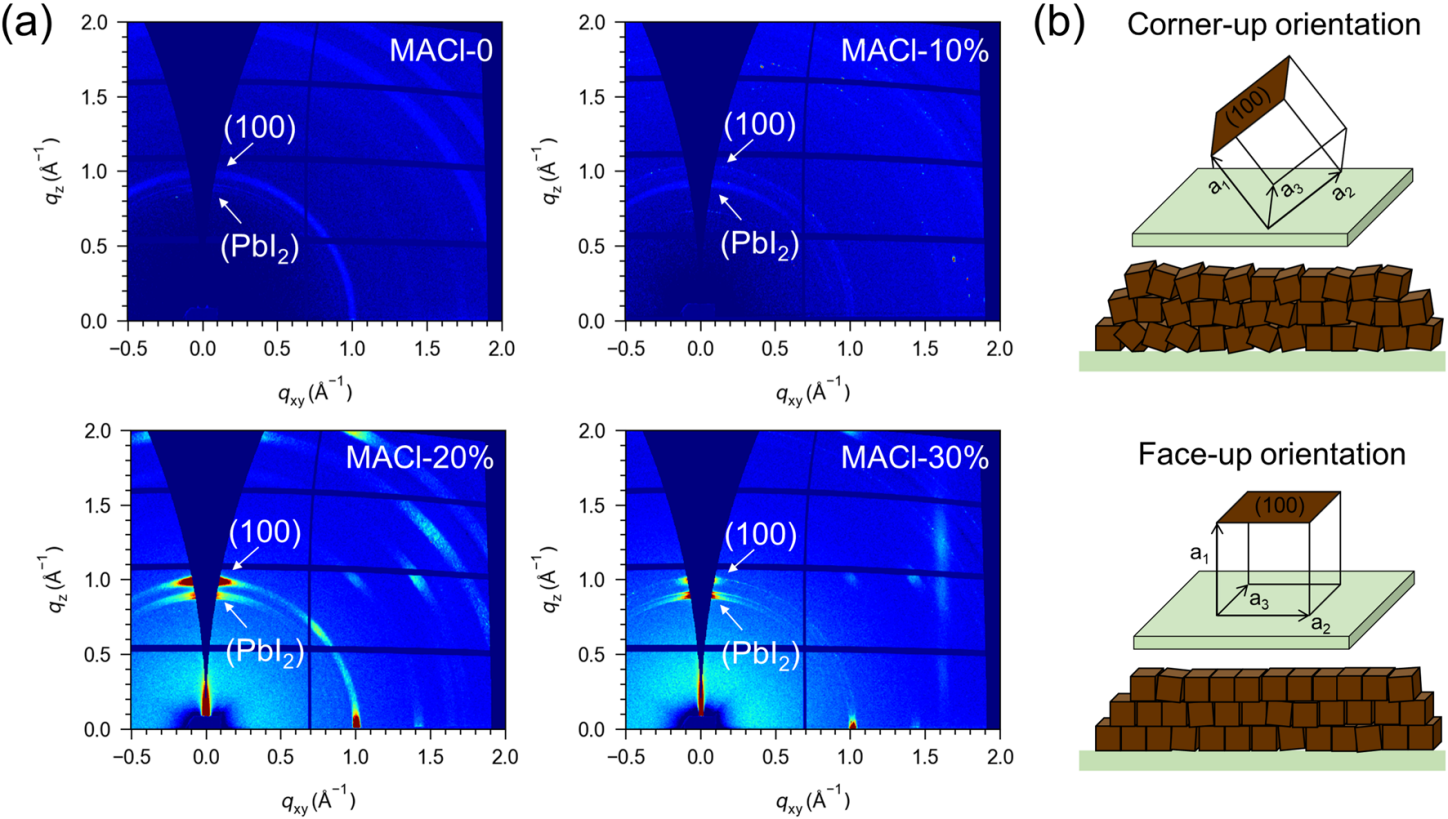
Crystal orientation of perovskite films fabricated via two-step doctor-blading with the different ratios of MACl. **a** Synchrotron-based 2D GIWAXS patterns. **b** A schematic illustration comparison between corner-up and face-up orientations with respect to the ITO substrate, where **a**_**1**_, **a**_**2**_, and **a**_**3**_ are lattice vectors

In order to investigate the crystallization and change of orientation of the perovskite crystals with or without MACl, the grazing-incidence wide-angle X-ray scattering (GIWAXS) is performed using synchrotron radiation on the different perovskite films. All the two-dimensional (2D) diffraction patterns are shown in Fig. 3a and have been processed using GIWAXS-Tools [33]. The Debye–Scherrer ring at the scattering vector *q* ≈ 1.0 Å^−1^ assigned to the (100) plane of *α*-phase perovskite and *q* ≈ 0.9 Å^−1^ corresponds to the (001) plane of PbI_2_. In 2D GIWAXS patterns, MACl-0 and MACl-10% exhibit weak diffraction rings and relatively uniform azimuthal intensity distribution, indicating irregular crystal orientation and weak crystallinity, in agreement with the above XRD and SEM discussed results. MACl-20% and MACl-30% films exhibit sharper, stronger, and more discrete diffraction spots, suggesting that the perovskite crystallites have a distinct preferred orientation. One-dimensional (1D) integration diagrams of 2D GIWAXS images are shown in Fig. S9. The 1D integrated intensity of MACl-20% is higher than the other samples, indicating the best surface crystallization. In addition, the PbI_2_ diffraction ring becomes more pronounced after adding MACl, probably released by the decomposition of the intermediate phase during the annealing process. Figure S10 displays the relative integration intensities along the (100) ring for perovskite films with various MACl ratios. It is observed that the MACl-0 perovskite film shows (100) diffraction peak with an azimuth angle (χ) located at approximately *χ* =  ± 55°, and the MACl-10% perovskite film exhibits (100) peak located at approximately *χ* =  ± 65°, corresponding to crystallites whose (100) plans are not entirely parallel to the substrate, resulting in a corner-up perovskite film orientation. On the other hand, the MACl-20% and MACl-30% perovskite films have strong (100) peaks located precisely at *χ* = 0° and approaching *χ* =  ± 90°, corresponding to crystallites whose (100) planes lie parallel and perpendicular to the substrate, thus producing a face-up orientation as shown in Fig. 3b [34]. The crystallographic orientation of the residual PbI_2_ changes in the same way as that of perovskite. The diffraction ring belonging to the PbI_2_ (001) plane shows a sharper peak at *χ* = 0° as the MACl ratio increases, indicating that the crystallization of PbI_2_ is also preferentially oriented (Fig. S11). It suggests that the face-up orientation of crystalline perovskites after MACl addition is related to the intermediate phase. MACl induces the organic salt to generate intermediate phases with PbI_2_ during the two-step deposition [35, 36]. During the subsequent perovskite growth, most intermediate phases act as a growth template and are converted to *α*-phase perovskite, while the remaining is decomposed into PbI_2_, resulting in a preferential orientation of both perovskite crystals and residual PbI_2_ perpendicular to the substrate. The highly ordered face-up domains can increase the charge-carrier effective mobilities and facilitate efficient charge transport in the devices [37], which is beneficial to the PSC performance.

**Fig. 4:**
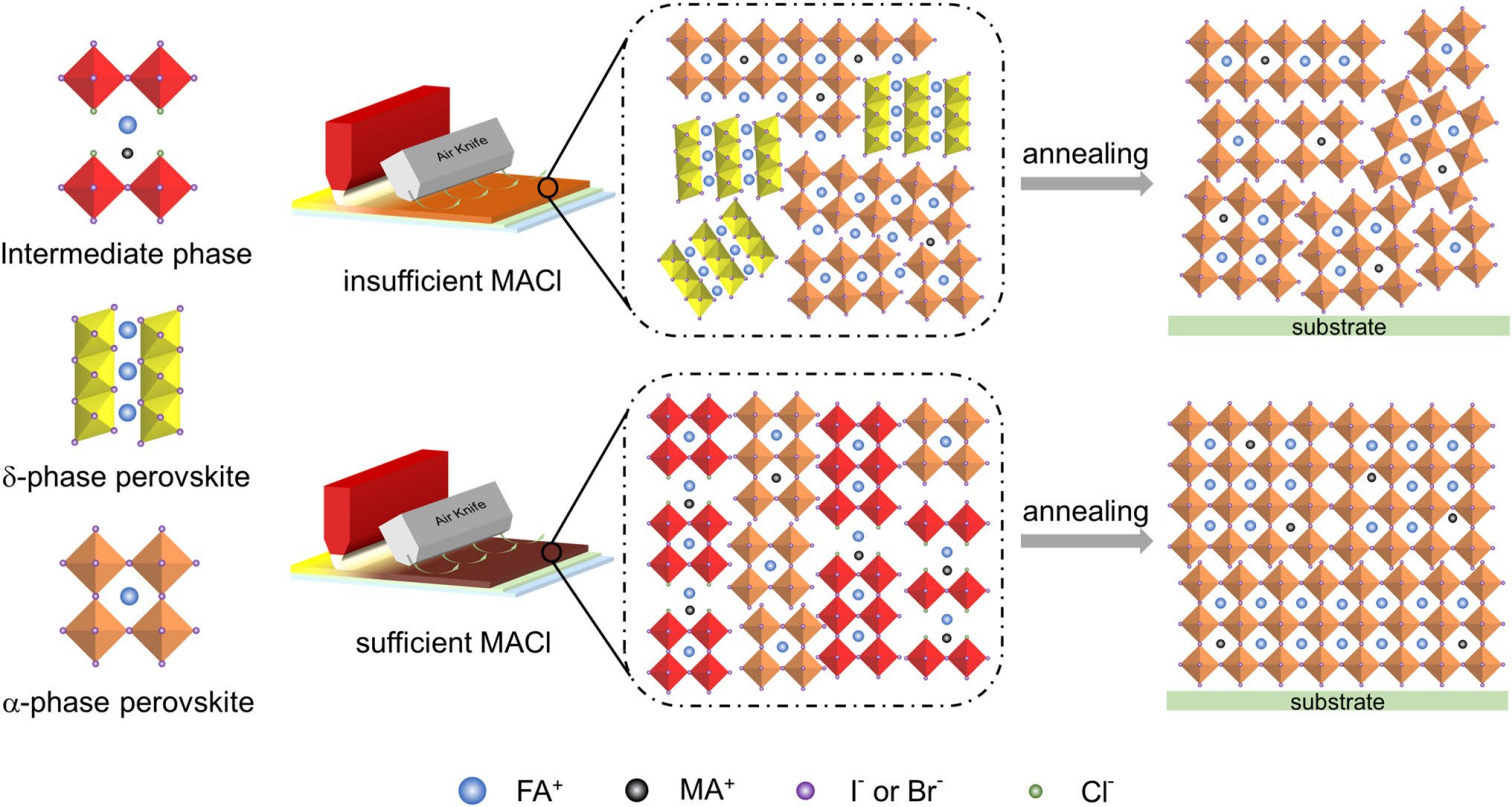
Schematic illustration of crystallization mechanism of perovskite films

Figure 4 shows schematic images comparing the crystallization processes of the perovskite with insufficient and sufficient MACl. The MACl-deficient perovskite precursor film exhibits the coexistence of *α*-phase and *δ*-phase, followed by a phase transition from *δ* to *α*-phase during thermal annealing. The phase transition process eventually leads to the collapse of the crystal volume, resulting in holes forming in the film, as shown in Fig. 1b. The low-dimensional intermediate phase in the MACl-sufficient perovskite precursor film provides a template for the growth of *α*-phase perovskite. This changes the nucleation and growth pathways to give a strong growth orientation and avoids the creation of internal cavities, which can help achieve high-quality perovskite films.

### Photovoltaic Performance of Two-Step Doctor-Bladed PSCs

**Fig. 5:**
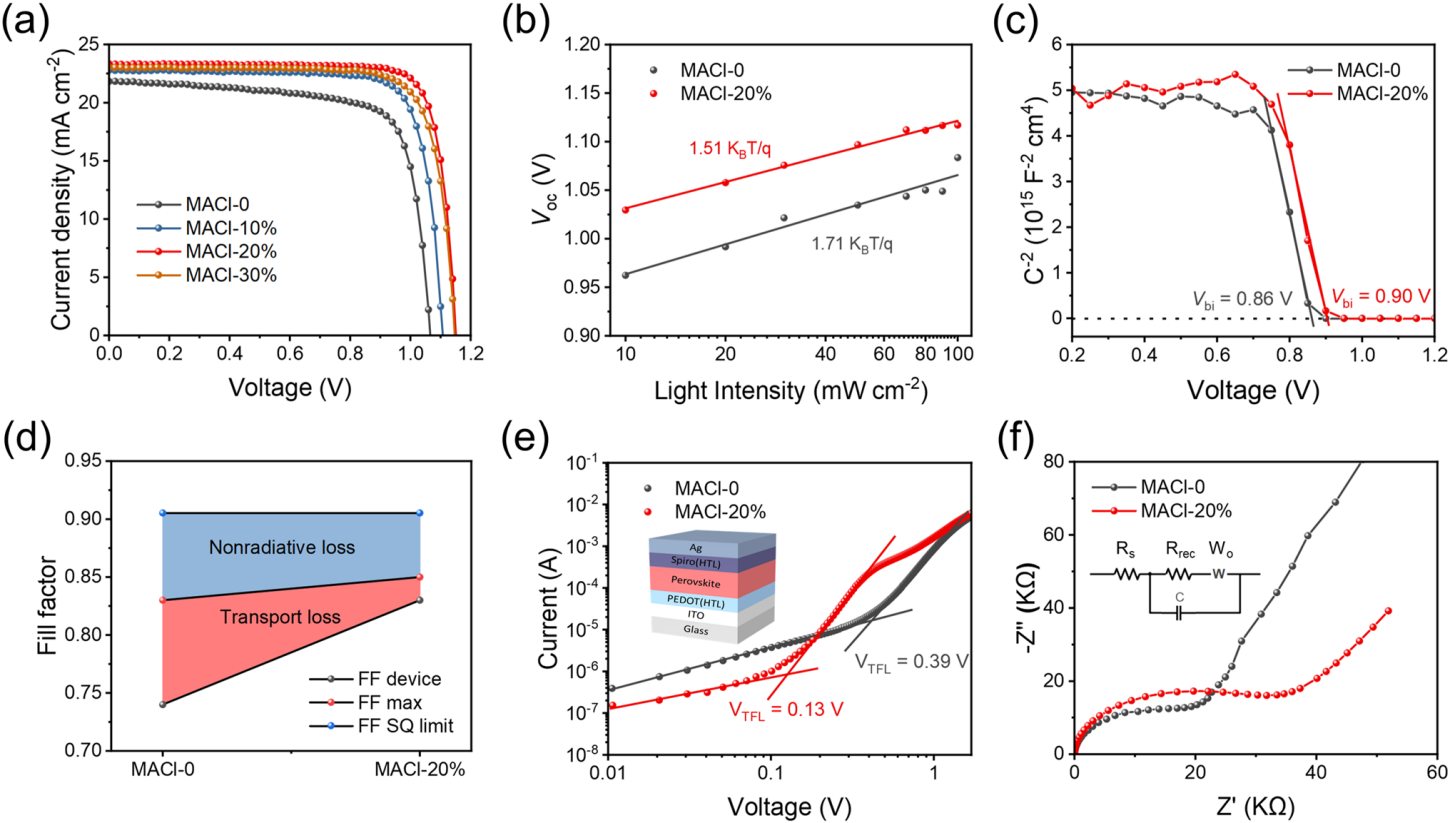
Photovoltaic performance and optoelectronic properties of PSCs with perovskite films fabricated via two-step doctor-blading with the different ratios of MACl. **a** *J–V* curves and corresponding parameters of typical PSCs. **b** Light intensity dependence of the *V*_oc_ of PSCs. **c** Mott–Schottky plots of the PSCs. **d** Fill factor limitation comprises nonradiative loss (blue area) and transport loss (pink area). **e** SCLC measurements of perovskite films. **f** Nyquist plots of EIS

PSCs are fabricated with a structure of ITO/SnO_2_/FA_1-*x*_MA_*x*_Pb(I_1-*y*_Br_*y*_)_3_/Spiro-OMeTAD/Ag, and the influence of MACl on photovoltaic performance is studied. Figure 5a shows the current density–voltage (*J–V*) curves of the typical devices with various MACl ratios. The detailed parameters with different MACl ratios, including the open-circuit voltage (*V*_oc_), short current density (*J*_sc_), fill factor (*FF*), and PCE, are summarized in Table S3. PSC with MACl-0 has a PCE of 17.32%, a *J*_sc_ of 21.84 mA cm^−2^, a *V*_oc_ of 1.06 V, and a *FF* of 74.47%. PSC with MACl-20% has a maximum PCE of 22.19% with a *J*_sc_ of 23.27 mA cm^−2^, a *V*_oc_ of 1.15 V, and a *FF* of 82.91%. The PCE of PSC with MACl-30% drops slightly to 20.96% due to the deterioration of the morphology. The photovoltaic metrics in Fig. S12 present the statistical photovoltaic parameters for the various MACl ratios devices fabricated independently under the same experimental conditions. The MACl-0 devices exhibit an average PCE of 16.62%, which is improved to 21.73% for the MACl-20% device. The change trends of device performance are consistent with the analysis of the perovskite film described above. Large grain size, better crystallinity, and longer carrier life give rise to better device performance.

For obtaining more information about charge transport and recombination mechanisms, the dependences of the *V*_oc_ on the light power *I* are determined, as shown in Fig. 5b. *V*_oc_ dependence on the light intensity provides information about recombination mechanisms [32, 38]. If the slope of *V*_oc_ versus *I* is close to *K*_B_*T*/*q*, where *T* is the temperature, *q* is the electron charge, and *K*_B_ is the Boltzmann constant, then bimolecular recombination is dominant. The slope greater than *K*_B_*T*/*q* indicates the presence of additional trap-assisted recombination [39, 40]. A reduction in the slope indicates a reduction in the additional recombination, which can improve the device’s performance [41]. In this case, the device with MACl-20% shows a weaker slope (1.51 *K*_B_*T*/*q*) than that of the MACl-0 device (1.71 *K*_B_*T*/*q*). It indicates a significantly inhibited trap-assisted nonradiative recombination in devices with MACl-20%, consistent with increases in *V*_oc_ and *FF*.

In addition to the suppressed nonradiative recombination, improved *V*_oc_ is associated with an elevation of the built-in potential (*V*_bi_). In Fig. 5c, Mott–Schottky plots are obtained from C–V measurement to estimate the *V*_bi_ and the driving force for photo-generated carriers. Compared with the MACl-0 device, the *V*_bi_ of the MACl-20% device is improved from 0.86 V to 0.90 V. The relatively high *V*_bi_ means the strengthened driving force for separating photo-generated charge carriers, which is conducive to enhancing the *V*_oc_ [42]. There is also a significant improvement in FF, from 74.47 to 82.91%, when the MACl ratio increases from 0 to 20%. The FF loss between the Shock–Queisser limit (FF_SQ_) and the measured FF from *J–V* curves consists of nonradiative loss and charge transport loss. The maximum FF (FF_max_) without charge transport loss can be calculated through Eq. (2) [43]:

2 $${\text{FF}}_{\max } = \frac{{\nu_{{{\text{oc}}}} - \ln \left( {\nu_{{{\text{oc}}}} + 0.72} \right)}}{{\nu_{{{\text{oc}}}} + 1}}, \nu_{{{\text{oc}}}} = \frac{{V_{{{\text{oc}}}} }}{{nk_{B} T/q}}$$

As shown in Fig. 5d, the increased FF for the MACl-20% mainly comes from the lower carrier transport loss, which is related to the higher crystallinity, more tightly packed grains, and more ordered orientation of perovskite. In addition, the suppressed carrier recombination, mainly nonradiative recombination, also contributes to the improvement of FF.

The above analysis shows that nonradiative recombination is significantly suppressed. Defects in perovskite films often act as nonradiative recombination centers for charge carriers. To further quantitatively investigate the trap density, hole-only devices are prepared and measured by the space-charge-limited-current (SCLC) method. As displayed in Fig. 5e, the trap-filled limited voltage (*V*_TFL_) is the transition voltage between Ohmic and trap-filling regions [44, 45]. The following equation can determine the trap-state density value (*N*_t_) [46]:

3 $$N_{t} = \frac{{2\varepsilon \varepsilon_{0} V_{{{\text{TFL}}}} }}{{eL^{2} }}$$

where *ε*_0_ is the vacuum dielectric constant, *ε* is the relative dielectric constant of perovskite, *e* is the elemental charge, and *L* is the thickness of the perovskite film. The defect density of MACl-20% perovskite (2.69 × 10^15^ cm^−3^) is appreciably lower than that of MACl-0 perovskite (8.08 × 10^15^ cm^−3^). The lower trap density is related to the pinhole-free and high-crystallinity perovskite film. In addition, electrochemical impedance spectroscopy (EIS) measurements in the dark are used to evaluate the carrier recombination kinetics. As shown in Fig. 5f, the recombination resistance (*R*_rec_) of the MACl-20% device (2.43 KΩ) is significantly higher than the MACl-0 device (1.89 KΩ), suggesting the reduced carrier recombination loss in PSCs, which increases the carrier transport and collection efficiency. The improvement in the *R*_rec_ implies an increase in the carrier recombination resistance, which could be attributed to the less trap-assisted nonradiative recombination in the device [47]. Furthermore, the MACl-20% devices have more excellent Warburg resistance (3.15 KΩ) than the MACl-0 device (1.16 KΩ), indicating suppressed ion migration [48].

**Fig. 6:**
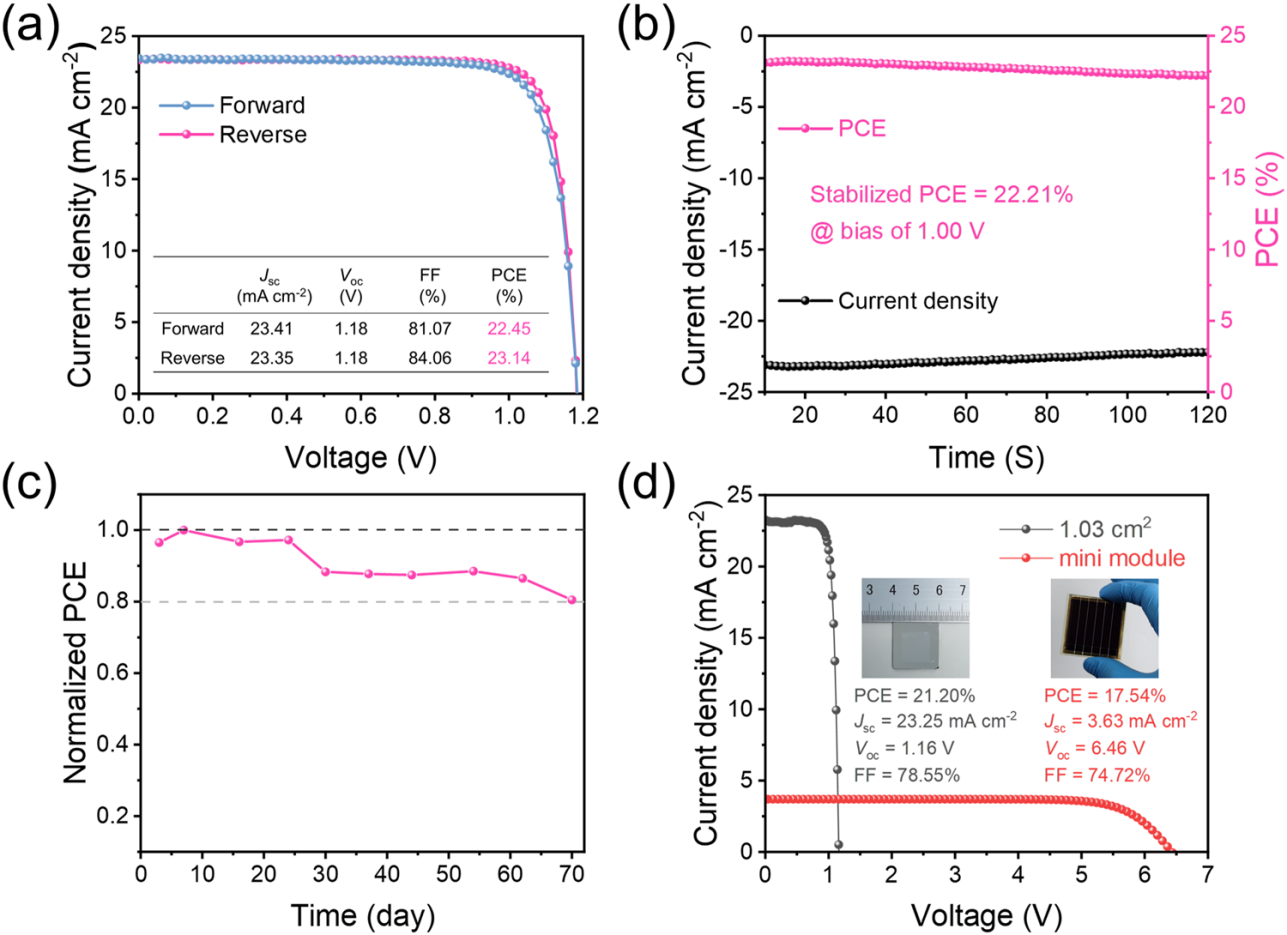
Performance of OAI-modified PSCs and mini-module. **a** *J–V* curves. **b** Stabilized power output measured at the maximum power point (MPP). **c** Long-term stability measurements of OAI-modified device without encapsulation under ambient conditions with about 30% relative humidity. **d** *J–V* curves of 1.03 cm^2^ PSCs and 10.93 cm^2^ mini-module. Insets are the pictures of 1.03 cm^2^ PSCs and 10.93 cm^2^ mini-module

Furthermore, octylammonium iodide (OAI) is used to passivate perovskite defects and fabricated PSCs with MACl-20%. OAI, a long-chained alkylammonium iodide, has been proven to form 2D perovskite and passivate surface defects to improve device efficiency and stability [49]. As shown in Fig. 6a, a champion PCE of 23.14% can be achieved with a *V*_oc_ of 1.18 V, *J*_sc_ of 23.35 mA cm^−2^, and FF of 84.08%. Compared with the results summarized in Table S4, it represents the published state of the art for photovoltaic devices by a two-step sequential doctor-blading or slot-die coating methods. Figure S13 shows the statistical graphs of various parameters of the OAI-modified PSCs, demonstrating the reproducibility of the device performance with a two-step doctor-blading. The integrated current density from the EQE spectra (Fig. S14) is 22.33 mA cm^−2^, which matched well with the *J*_sc_ values in the *J–V* curve. The stabilized PCE outputs of the devices are further monitored at bias voltages (1.0 V) of maximum power point (MPP) and exhibit a stable output PCE of 22.21%, as shown in Fig. 6b. Furthermore, the long-term stability is tested, and the unencapsulated PSC modified by OAI can retain 80% of its initial PCE after storage in ambient conditions for 70 days under about 30% relative humidity (Fig. 6c).

With the rapid leap in the efficiency of PSCs, developing large-area PSCs is an inevitable path from laboratory to commercialization. We fabricated large-area PSCs with active areas of 1.03 cm^2^ using a two-step sequential doctor-blading strategy. As shown in Fig. 6d, the best-performance large-area device shows *V*_oc_, *J*_sc_, and *FF* values of 1.16 V, 23.26 mA cm^−2^, and 78.55%, respectively, yielding a PCE of 21.20%. The high PCE in a large-area device could be attributed to the high-quality perovskite film with large grain size, strong orientation, low defect density, and low charge transport resistance. Given the excellent performance of large-area devices, the mini-modules consisting of six sub-cells are fabricated with an active area of 10.93 cm^2^. As expected, the PSC mini-modules achieve a PCE of 17.54% with *V*_oc_ of 6.46 V, *J*_sc_ of 3.63 mA cm^−2^, and FF of 74.72%. Compared with small-area devices, the reduced *J*_sc_ and FF could be attributed to the large series resistance caused by the longer carrier transport paths [50].

### Stability of Perovskite Films

In addition, the intrinsic stability of perovskite films with the different MACl ratios of films without OAI treatment is studied by storing them in ambient conditions with a humidity of 40% RH. It is found that perovskite films with MACl-30% and MACl-20% are still able to maintain the black *α*-phase perovskite, while perovskite film with MACl-10% mainly changes to yellow phase. Perovskite film with MACl-0 is even worse and completely changes to a yellow non-photoactive phase, as shown in Fig. S15. Comparing the XRD patterns of fresh and aged perovskite films (Fig. S16), it is found that the diffraction spectra of perovskite films with MACl-30% and MACl-20% are unchanged, and no excess diffraction peaks appear. But perovskite film with MACl-10% shows a new diffraction peak at 11.78°, corresponding to *δ*-phase perovskite [51], and the *δ*-phase diffraction peak is stronger than *α*-phase perovskite, indicating that most of the *α*-phase perovskite is converted to *δ*-phase. The diffraction peak of *α*-phase perovskite of MACl-0 disappears and is replaced by the diffraction peak of *δ*-phase. The enhanced stability of perovskite film can be attributed to the conversion of higher film quality with large grains, denser films, and stronger ordered orientations.

## Conclusions

In summary, we exploited the modulating effect of MACl on the perovskite growth to fabricate high-quality, strongly oriented perovskite films using a two-step sequential doctor-blading process. Consequently, the perovskite layer with a larger grain size reduces grain boundaries, enhanced crystallinity, low defect density, and preferential face-up orientation, inhibiting nonradiative recombination and enhancing the transmission of charge carriers. As expected, it can achieve a high PCE of 23.14%, and the unencapsulated device sustained 80% of its initial PCE after 70 days under a relative humidity of 30% in ambient conditions. The large-area device (1.03 cm^2^) and mini-module (10.93 cm^2^) achieve PCEs of 21.20% and 17.54%, respectively, based on uniform large-scale perovskite films. This work provides a strategy to fabricate large-area high-quality perovskite films in ambient air and has instructive significance for realizing the industrial production of PSCs.

### Supplementary Information

Below is the link to the electronic supplementary material.

Supplementary file1 (PDF 1647 kb)
