# Supplementary material for: Crystallization and Orientation Modulation Enable Highly Efficient Doctor-Bladed Perovskite Solar Cells
Source: Nanomicro Lett. 2023 Jun 29;15:164. doi: 10.1007/s40820-023-01138-x (PMC10310680; doi:10.1007/s40820-023-01138-x)
Supplement: Supplementary file 1 — Supplementary file1 (PDF 1647 kb) [file 40820_2023_1138_MOESM1_ESM.pdf]

Supporting Information for

Crystallization and Orientation Modulation Enable Highly Efficient Doctor-Bladed Perovskite Solar Cells

Jianhui Chang[1], Erming Feng[1], Hengyue Li[1], Yang Ding[1], Caoyu Long[1], Yuanji Gao[1], Yingguo Yang[2], Chenyi Yi[3], Zijian Zheng[4], Junliang Yang[1,*]

[1] Hunan Key Laboratory of Nanophotonics and Devices, School of Physics and Electronics, Central South University, Changsha, Hunan 410083, P. R. China

[2] Shanghai Synchrotron Radiation Facility (SSRF), Shanghai Advanced Research Institute & Chinese Academy of Sciences, Shanghai 201204, P. R. China

[3] Department of Electrical Engineering, Tsinghua University, Beijing 100084, P. R. China

[4] Department of Applied Biology and Chemical Technology, Faculty of Science, The Hong Kong Polytechnic University, Hong Kong SAR 999077, P. R. China

*Corresponding author E-mail: junliang.yang@csu.edu.cn (Junliang Yang)

Supplementary Figures and Tables

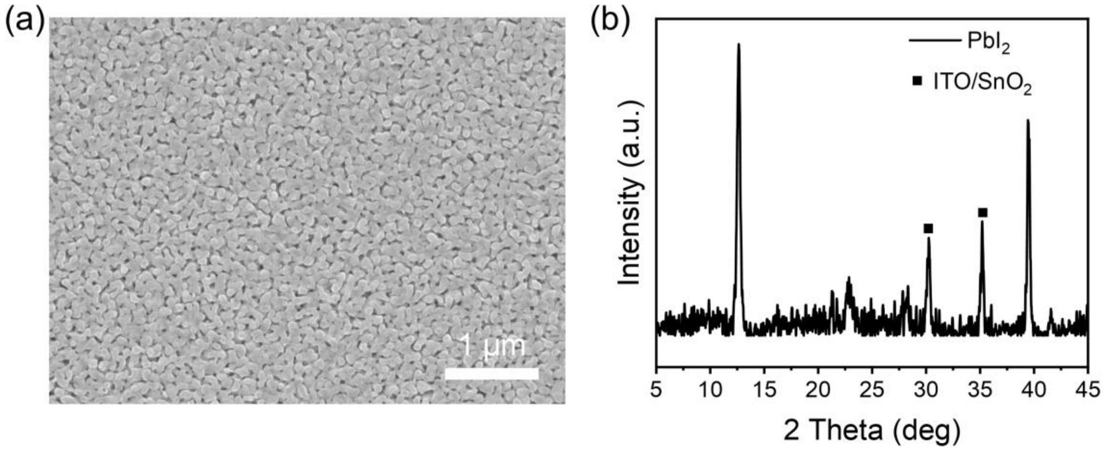

Fig. S1 a SEM image of the PbI_2 film deposited by the doctor-blading. **b** XRD pattern of the PbI_2 film

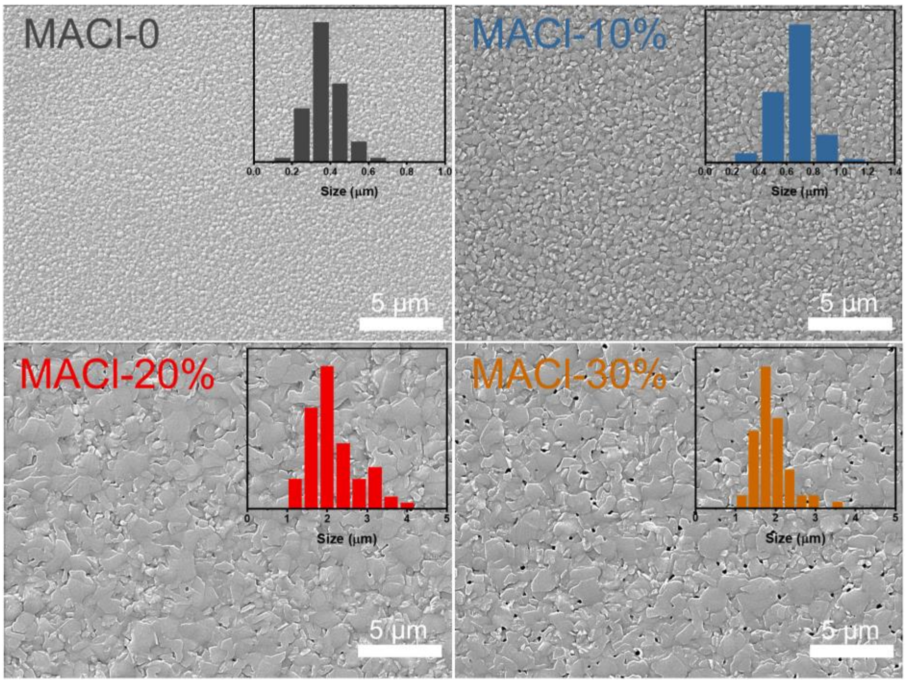

Fig. S2 SEM images of perovskite films at 10000 × magnification. The insets show the distribution of particle grain sizes

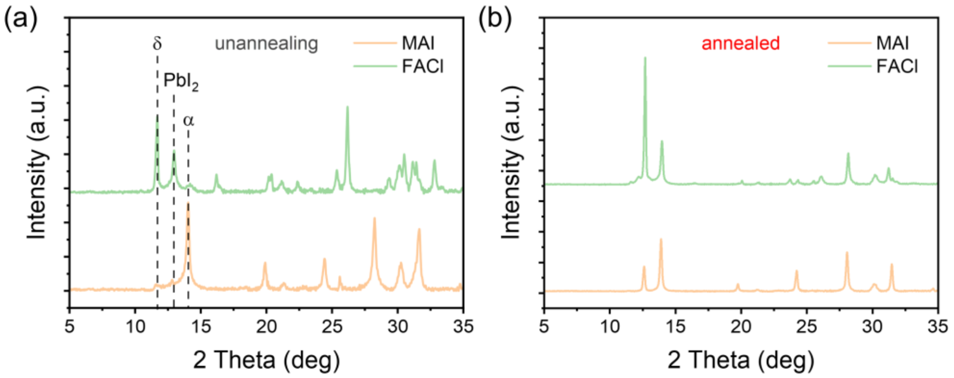

Fig. S3 a XRD patterns of the unannealed perovskite films with adding MAI or FACl. **b** XRD patterns of the annealed perovskite films with adding MAI or FACl

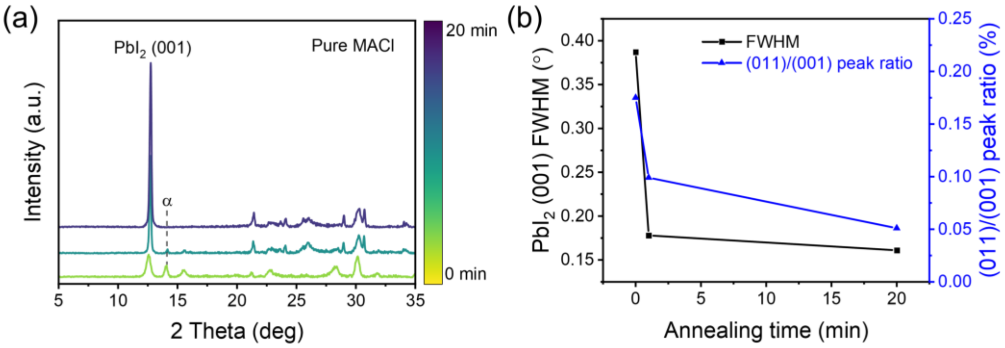

Fig. S4 a XRD patterns of pure MACl with various annealing times. **b** FWHM of (001) peak and a (011) to (001) diffraction peak ratio belonging to PbI_2 extracted from the XRD patterns of pure MACl with various annealing times

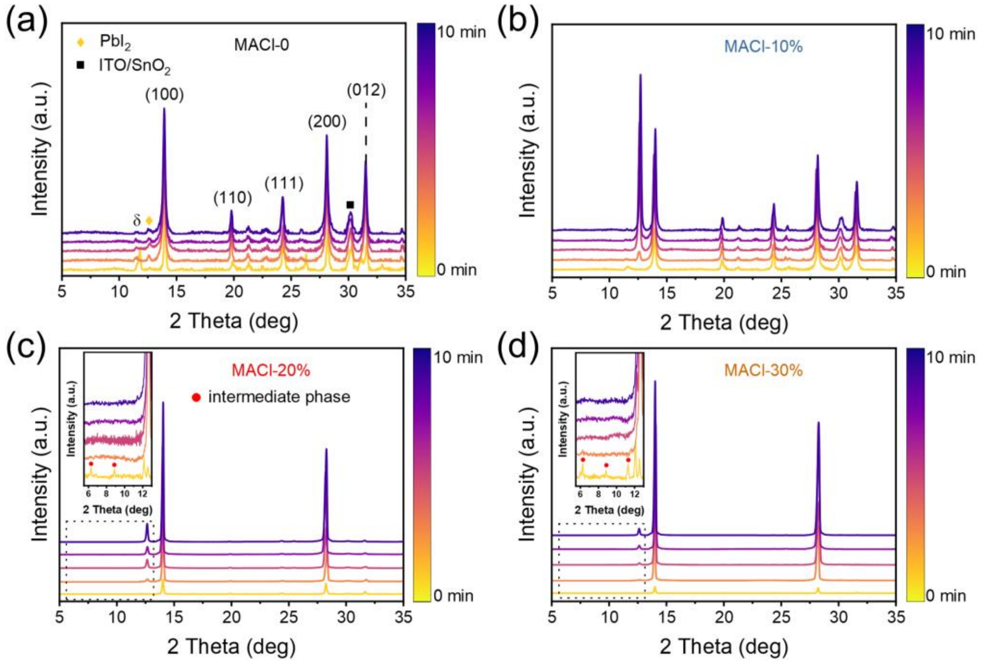

Fig. S5 XRD patterns of **a** MACl-0, **b** MACl-10%, **c** MACl-20%, and **d** MACl-30% with various annealing times

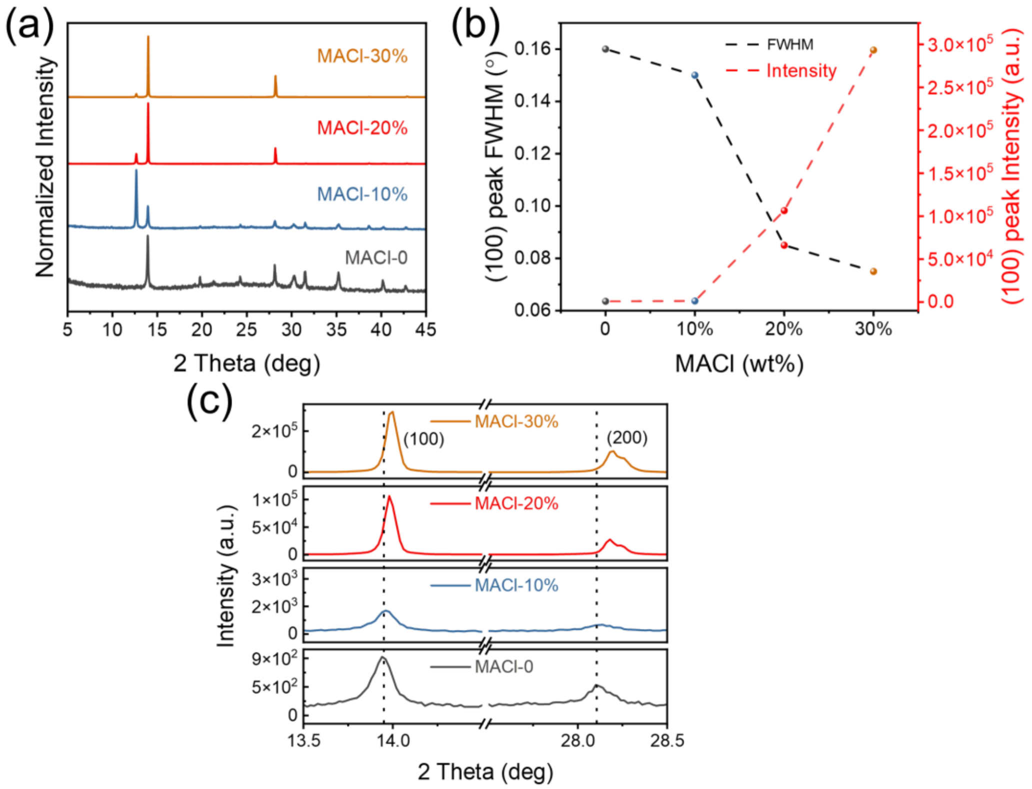

Fig. S6 a Normalized XRD patterns of the annealed films with various ratios of MACl. **b** FWHM and intensity of (100) peak with various ratios of MACl. **c** XRD diffraction peaks of (100) and (200) crystal planes for perovskite films with the different MACl ratios. With increasing the MACl ratio, the diffraction peak of (100) and (200) perovskite crystal plane gradually shifts to high diffraction angle because of the increased proportion of small-sized MA^+ in the perovskite phase

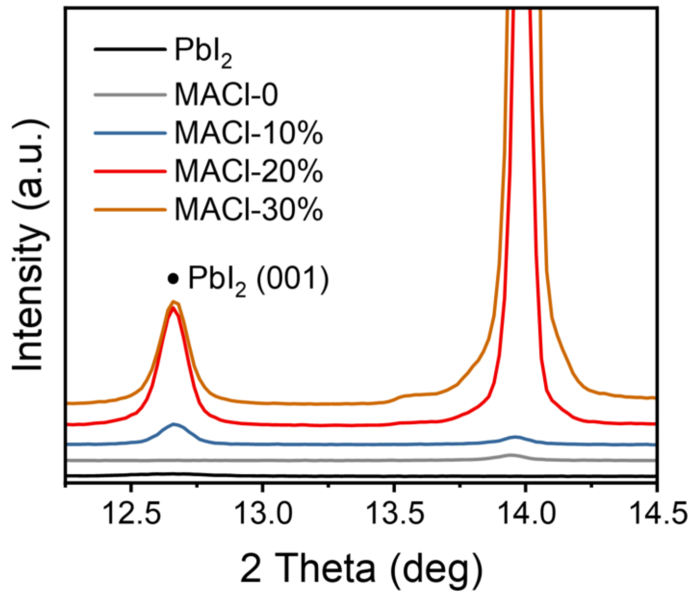

Fig. S7 Characteristic XRD diffraction peaks of PbI_2. The intensity of PbI_2 diffraction peaks increases with the MACl ratio

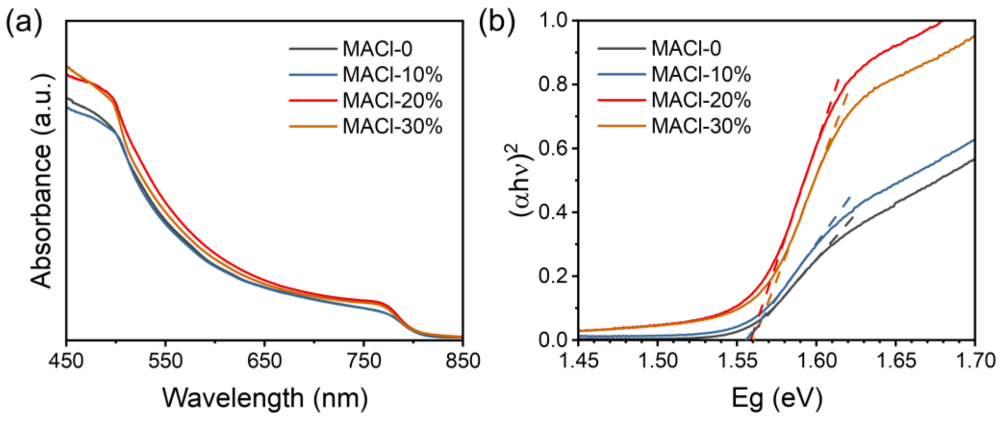

Fig. S8 **a** UV-Vis absorbance spectra. **b** Tauc plots of the perovskite films with the different MACl ratios

Table S1 Band gap was extracted from the Tauc-plot

	MACl-0	MACl-10%	MACl-20%	MACl-30%
E_g (eV)	1.556	1.556	1.558	1.561

Table S2 PL carrier lifetimes were extracted from the TRPL decay measurements

	A_1	τ_1 (ns)	A_2	τ_2 (ns)	A_3	τ_3 (ns)	τ_{ave} (ns)
MACl-0	0.15	15.75	0.59	88.26	0.22	300.98	203.52
MACl-10%	0.21	21.60	0.54	112.22	0.21	401.86	271.80
MACl-20%	0.10	23.60	0.58	131.03	0.30	462.93	342.78
MACl-30%	0.28	32.23	0.56	114.76	0.17	451.04	281.22

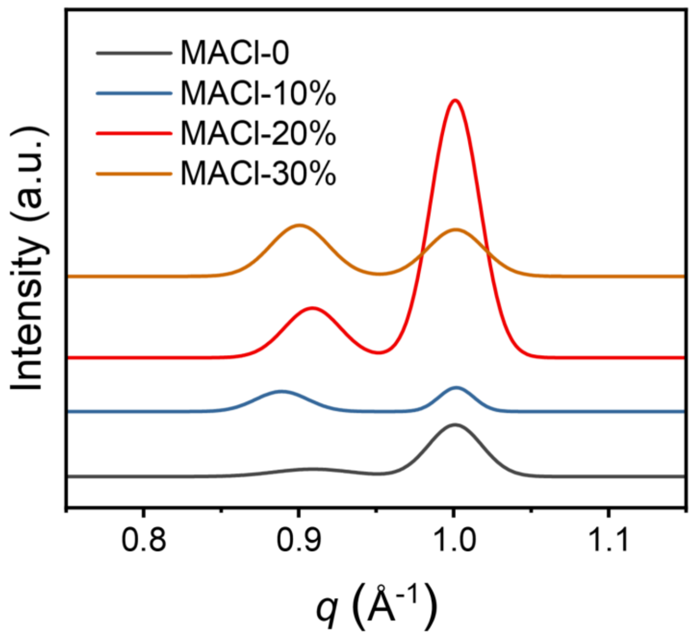

Fig. S9 Angular-integrated diffraction patterns of the perovskite films calculated from the 2D GIWAXS patterns

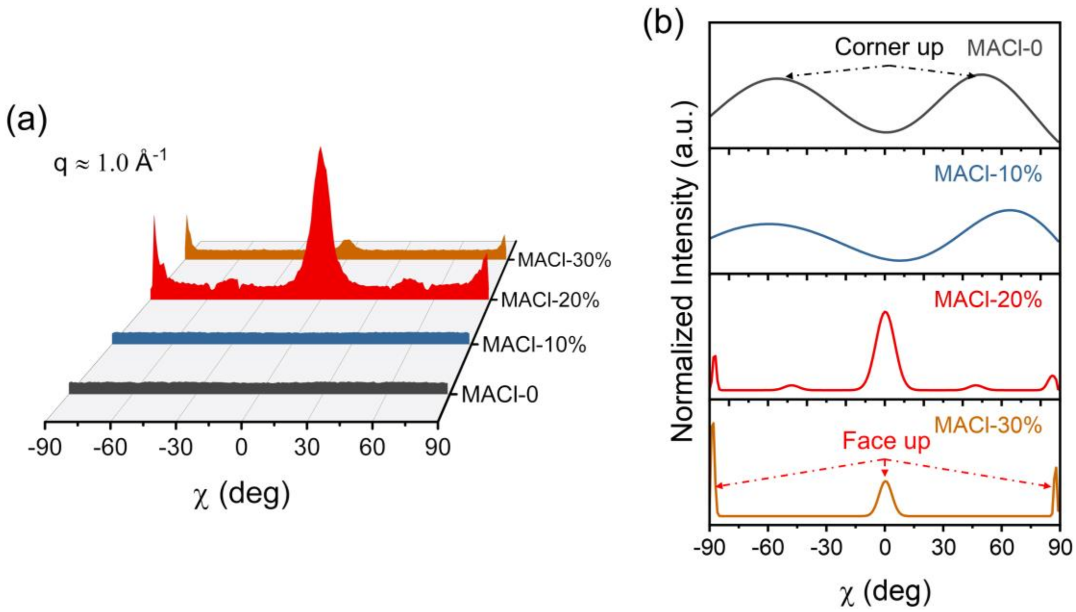

Fig. S10 a Azimuth integration at q ≈ 1.0 $Å^{-1}$ for perovskite films obtained from 2D GIWAXS patterns. **b** The (100) peak as a function of the azimuth angle (χ) for a series of perovskite films with various ratios of MACl. The analysis shows a corner-up orientation for the low MACl ratio, but a predominant face-up orientation for films with 20% or more excess MACl

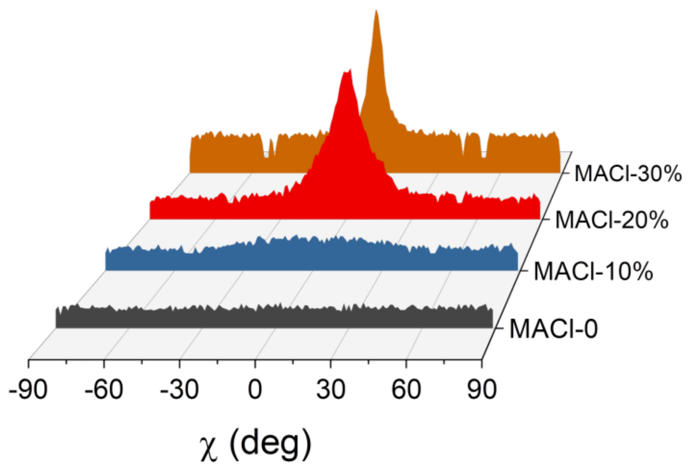

Fig. S11 Azimuth integration at q ≈ 0.9 $Å^{-1}$ (represent PbI_2) for perovskite films

Table S3 Summary of photovoltaic parameters devices with different MACl ratios (**Fig.** 4a)

	J_{sc} (mA cm^{-2})	V_{oc} (V)	FF (%)	PCE (%)	Ave. PCE (%)
MACl-0	21.84	1.06	74.47	17.32	16.62±0.49
MACl-10%	22.89	1.11	79.77	20.09	19.25±0.67
MACl-20%	23.27	1.15	82.91	22.19	21.73±0.26
MACl-30%	23.04	1.14	79.50	20.96	20.28±0.37

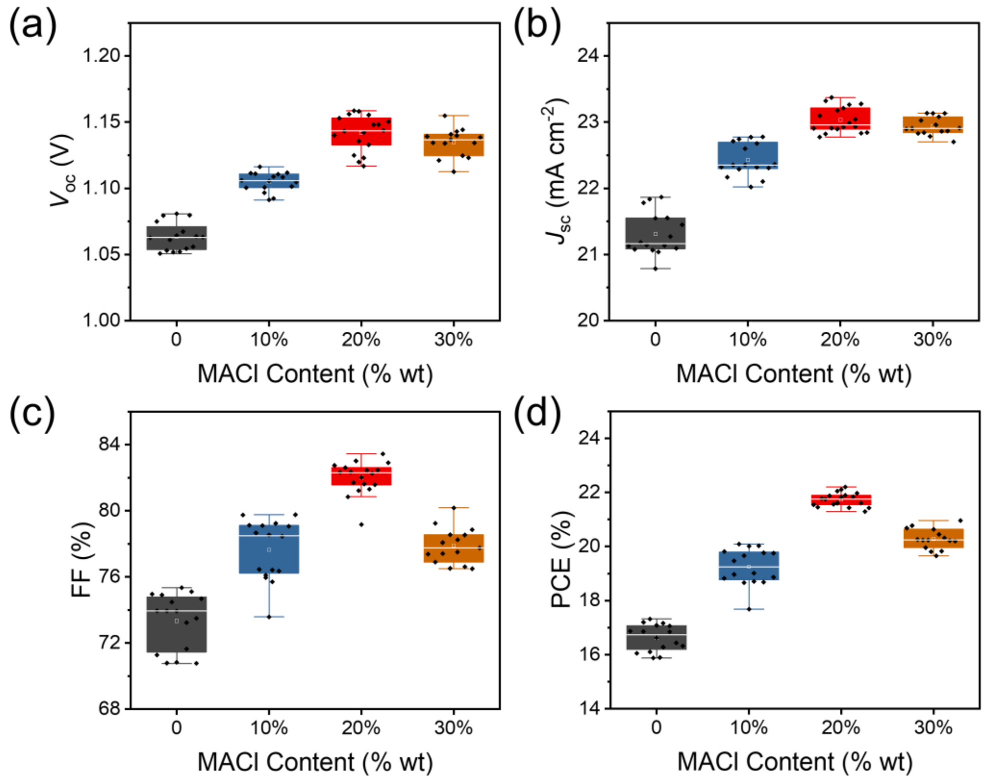

Fig. S12 Statistics of photovoltaic parameters (J_{sc}, V_{oc}, FF, and PCE, respectively) of PSCs with different MACl ratios

Table S4 A summary PCEs for PSCs prepared by two-step doctor blading or two-step slot die

Year	Structures	Techniques	PCE	Refs
2019	ITO/NiO_x/$MAPbI_3$/PCBM/BCP/ Ag	two-step doctor blading	16.71	[S1]
2020	FTO/SnO_2/$(FAPbI_3)_{1-x}(MAPbBr_3)_x$/ Spiro-OMeTAD/Au	two-step doctor blading	20.49	[S2]
2020	ITO/NiO_x-NPs/$CH_3NH_3PbI_3$/ $PC_{61}BM$/BCP/Ag	two-step doctor blading	19.01	[S3]
2020	ITO/NiO_x/$FAPbI_3$/$PC_{61}BM$/BCP/ Ag	two-step doctor blading	18.41	[S4]
2021	ITO/PTAA/$MAPbI_3$/PCBM/BCP/Ag	two-step doctor blading	20.33	[S5]
2022	FTO/SnO_2/$FA_xMA_{1-x}PbI_3$/ Spiro-OMeTAD/Au	two-step doctor blading	22.77	[S6]
2021	FTO/c-TiO_2/SnO_2/$CsFA_{1-x}MA_xPbI_{1-y}Br_y$/ Spiro-OMeTAD/Au	two-step slot die	19.00	[S7]
2022	ITO/SnO_2/$Cs_{0.05}MA_{0.4}FA_{0.55}Pb(I_{0.96}Br_{0.04})_3$/ Spiro-OMeTAD/Au	two-step slot die	19.20	[S8]
2022	ITO/SnO_2/$CsFA_{1-x}MA_xPbI_{1-y}Br_y$/ Spiro-OMeTAD/Ag	two-step slot die	18.13	[S9]
2023	ITO/SnO_2/$FA_{1-x}MA_xPbI_{1-y}Br_y$/ Spiro-OMeTAD/Ag	two-step doctor blading	23.14	This work

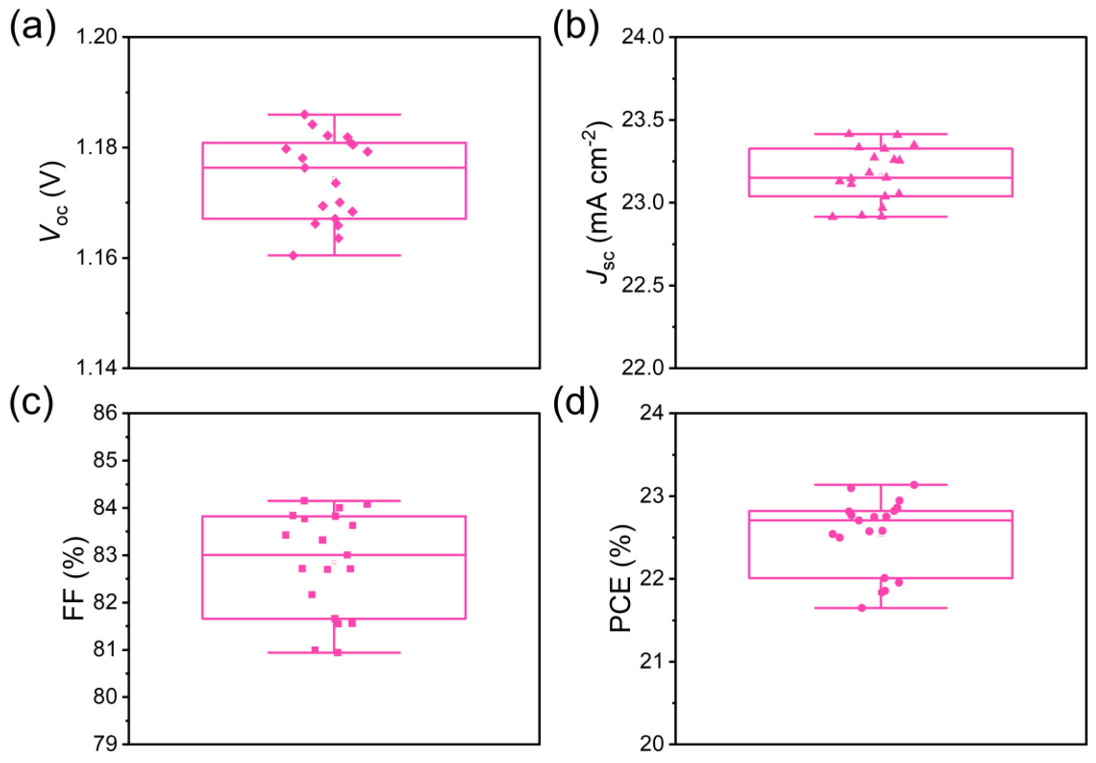

Fig. S13 Statistics of photovoltaic parameters of PSCs (J_{sc}, V_{oc}, FF, PCE, respectively) after OAI modification

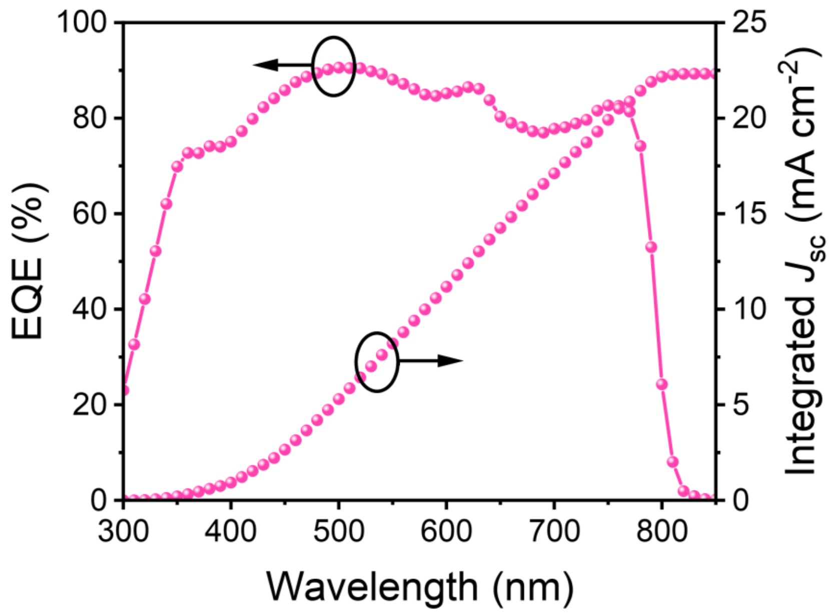

Fig. S14 EQE spectra and corresponding integrated J_{sc} of the OAI-modified PSC

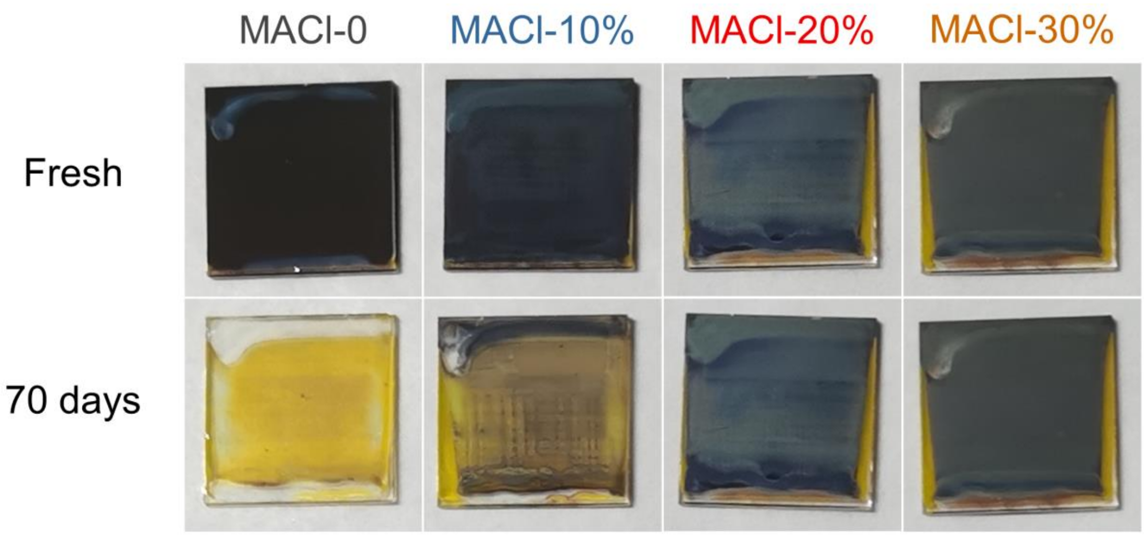

Fig. S15 The images of perovskite films with different MACl ratios. The top images are fresh perovskites, and the bottom images are the perovskites after 70 days of aging in ambient conditions under a humidity of 40% RH

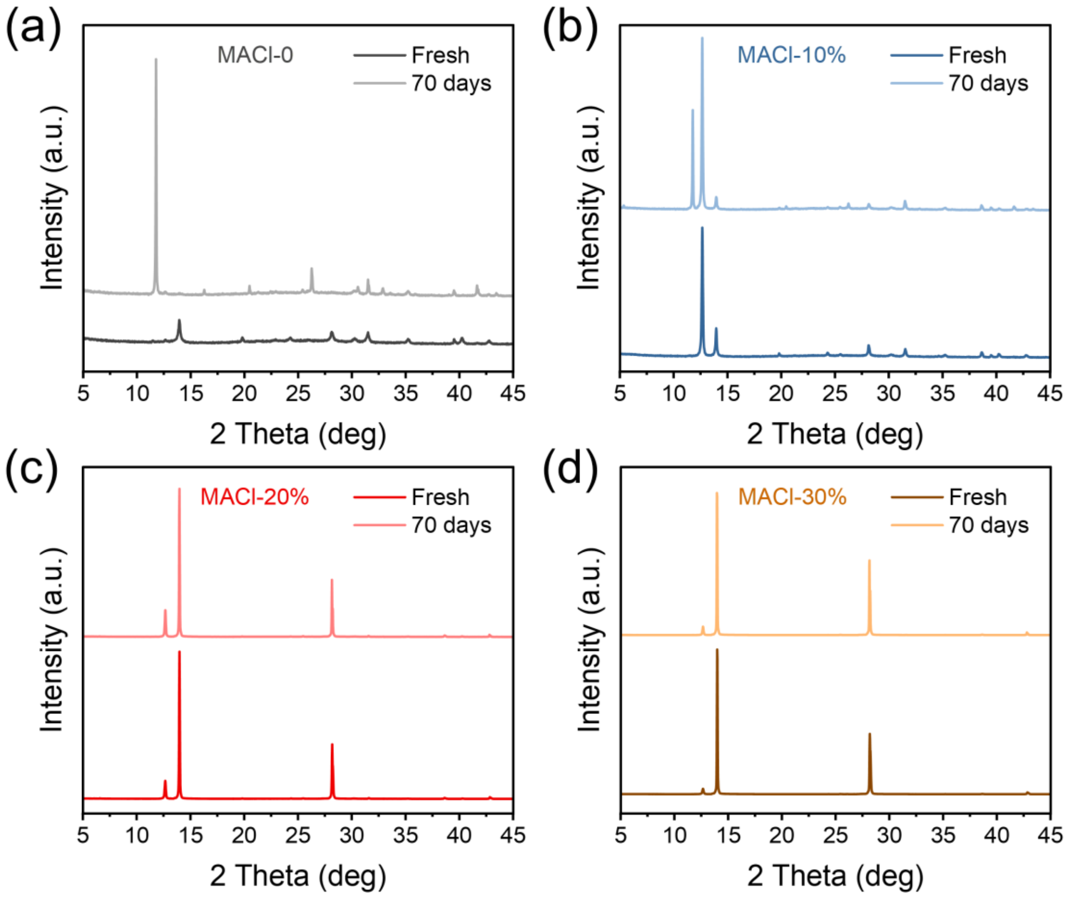

Fig. S16 XRD patterns of fresh and aged perovskite films under 40% RH. **a** MACl-0. **b** MACl-10%. **c** MACl-20%. **d** MACl-30%

Supplementary Figures

[S1]F. Guo, W. He, S. Qiu, C. Wang, X. Liu, et al. Sequential deposition of high-quality photovoltaic perovskite layers via scalable printing methods. Adv. Funct. Mater. **29**(24), 1900964 (2019). https://doi.org/10.1002/adfm.201900964

[S2]J. Zhang, T. Bu, J. Li, H. Li, Y. Mo, et al. Two-step sequential blade-coating of high quality perovskite layers for efficient solar cells and modules. J. Mater. Chem. A **8**(17), 8447–8454 (2020). https://doi.org/10.1039/D0TA02043E

[S3]Z. Huang, X. Hu, Z. Xing, X. Meng, X. Duan, et al. Stabilized and operational pbi_2 precursor ink for large-scale perovskite solar cells via two-step blade-coating. J. Phys. Chem. C **124**(15), 8129–8139 (2020). https://doi.org/10.1021/acs.jpcc.0c00908

[S4]W. He, J. Hu, C. Chen, Y. Chen, L. Zeng, et al. Temperature-assisted crystal growth of photovoltaic α-phase $FAPbI_3$ thin films by sequential blade coating. ACS Appl. Mater. Interfaces 12(50), 55830–55837 (2020). https://doi.org/10.1021/acsami.0c15733

[S5]J. Li, X. Meng, Z. Huang, R. Dai, W. Sheng, et al. A regularity-based fullerene interfacial layer for efficient and stable perovskite solar cells via blade-coating. Adv. Funct. Mater. **32**(1), 2105917 (2021). https://doi.org/10.1002/adfm.202105917

[S6]W. Yu, J. Li, X. Gao, C. Tian, H. Zhu, et al. Two-step sequential blade-coating large-area fa-based perovskite thin film via a controlled PbI_2 microstructure. Acta Phys. -Chim. Sin. **39**(2), 2203048 (2023). https://doi.org/10.3866/PKU.WHXB202203048

[S7]I. Zimmermann, M. Al Atem, O. Fournier, S. Bernard, S. Jutteau, et al. Sequentially Slot-Die-Coated Perovskite for Efficient and Scalable Solar Cells. Adv. Mater. Interfaces **8**(18), 2100743 (2021). https://doi.org/10.1002/admi.202100743

[S8]I. Zimmermann, M. Provost, S. Mejaouri, M. Al Atem, A. Blaizot, et al. Industrially compatible fabrication process of perovskite-based mini-modules coupling sequential slot-die coating and chemical bath deposition. ACS Appl. Mater. Interfaces **14**(9), 11636–11644 (2022). https://doi.org/10.1021/acsami.1c24558

[S9]H. Li, C. Zuo, D. Angmo, H. Weerasinghe, M. Gao, et al. Fully roll-to-roll processed efficient perovskite solar cells via precise control on the morphology of PbI_2:CsI layer. Nano-Micro Lett. **14**, 79 (2022). https://doi.org/10.1007/s40820-022-00815-7
